# Association between nonalcoholic fatty liver disease and incident diabetes mellitus among Japanese: a retrospective cohort study using propensity score matching

**DOI:** 10.1186/s12944-021-01485-x

**Published:** 2021-06-15

**Authors:** Xiaodan Zheng, Changchun Cao, Yongcheng He, Xinyu Wang, Jun Wu, Haofei Hu

**Affiliations:** 1 Department of Neurology, Peking University Shenzhen Hospital, Shenzhen, 518000 Guangdong Province China; 2 Department of Clinical Medicine, Shantou University Medical College, Shantou, 515000 Guangdong Province China; 3 Department of Rehabilitation, Shenzhen Dapeng New District Nan’ao People’s Hospital, Shenzhen, 518000 Guangdong Province China; 4 Department of Nephrology, Shenzhen Hengsheng Hospital, Shenzhen, 518000 Guangdong Province China; 5 Department of Endocrinology, The First Affiliated Hospital of Shenzhen University, Shenzhen, 518000 Guangdong Province China; 6 Department of Nephrology, The First Affiliated Hospital of Shenzhen University, Shenzhen, 518000 Guangdong Province China

**Keywords:** Nonalcoholic fatty liver disease, Diabetes mellitus, Propensity-score matching, Inverse probability of treatment weights, Cox proportional hazards regression, Sensitivity analysis

## Abstract

**Background:**

Previous studies have demonstrated that nonalcoholic fatty liver disease (NAFLD) is a significant risk factor for diabetes mellitus (DM). However, these studies did not completely determine the relationship between NAFLD and DM due to unbalanced confounding factors. The propensity score (PS) is the conditional probability of having a particular exposure, given a set of baseline measured covariates. Propensity score matching (PSM) analysis could minimise the effects of potential confounders. Thus, this study aimed to use PSM analysis to explore the association between NAFLD and DM in a large Japanese cohort.

**Methods:**

This retrospective PSM cohort study was performed on 14,280 Japanese participants without DM at baseline in Murakami Memorial Hospital between 2004 and 2015. The independent variable was NAFLD at baseline, and the outcome was the incidence of DM during follow-up. One-to-one PSM revealed 1671 participants with and without NAFLD. A doubly robust estimation method was applied to verify the correlation between NAFLD and DM.

**Results:**

The risk of developing DM in participants with NAFLD increased by 98% according to the PSM analysis (HR = 1.98, 95% confidence interval [CI]: 1.41–2.80, *P* < 0.0001). The risk of developing DM in the NAFLD participants was 2.33 times that of the non-NAFLD participants in the PSM cohort after adjusting for the demographic and laboratory biochemical variables (HR = 2.33, 95% CI: 1.63–3.32, *P* < 0.0001). The participants with NAFLD had a 95% increased risk of DM after adjusting for PS (HR = 1.95, 95% CI: 1.39–2.75, *P* = 0.0001). All potential confounding variables were not significantly associated with NAFLD and DM after PSM in the subgroup analysis. In the sensitivity analysis, the participants with NAFLD had a 2.17-fold higher risk of developing DM in the original cohort (HR = 2.17, 95% CI: 1.63–2.88, *P* < 0.0001) and were 2.27-fold more likely to develop DM in the weighted cohort (HR = 2.27, 95% CI: 1.91–2.69, *P* < 0.00001).

**Conclusions:**

NAFLD was an independent risk factor for the development of DM. The risk of developing DM in the NAFLD participants was 2.33 times that of the non-NAFLD participants in the PSM cohort after adjusting for the demographic and laboratory biochemical variables. The participants with NAFLD had a 95% increased risk of DM after adjusting for PS.

## Introduction

Diabetes mellitus (DM) has become a serious global public health problem. According to international epidemiological research on DM, the prevalence of DM in 2019 was 9.3% (approximately 500 million people) [1]. DM and its complications can seriously affect the health of patients and increase medical costs, which can lead to a heavy economic burden on the patients and society [2]. DM is a metabolic disease characterised by hyperglycaemia caused by insufficient insulin secretion or insulin resistance (IR) [3]. Many researchers have explored the pathogenesis and risk factors of DM.

Some prospective cohort studies recently reported that nonalcoholic fatty liver disease (NAFLD) is a significant risk factor for DM [4, 5]. NAFLD is often accompanied by DM, obesity, and hyperlipidaemia [6, 7]. Additionally, some studies found that NAFLD was an independent risk factor for DM after adjusting for confounding variables [8, 9]. A recent meta-analysis of 33 studies involving more than 500,000 individuals showed that participants with NAFLD had a 1.19-fold higher risk of developing DM than participants without NAFLD [10].

The propensity score (PS) is defined as the conditional probability of having a particular exposure (NAFLD versus non-NAFLD), given a set of baseline measured covariates. The propensity score matching (PSM) method is useful in studies in which there are many covariates potentially confounding a rare outcome, there is potential confounding by indication, and there are resource constraints that prevent the conduction of randomized clinical trials. Given the numerous potential confounding variables, a traditional parsimonious regression model could result in bias due to unmeasured or residual confounding, whereas the inclusion of more variables could result in overfitting of the model, potentially preventing identification of the association between the exposure of interest and the outcome [11]. Therefore, PSM analysis was used in this study to explore the actual association between NAFLD and DM in the NAGALA (NAfld in the Gifu Area, Longitudinal Analysis) database of 14,280 Japanese people.

## Methods

### Study design and data source

This was a secondary retrospective study based on NAGALA, sourced from the public DRYAD database (www.Datadryad.org.database). Raw data were provided by Okamura et al. [12]. The original study included 20,944 participants who underwent medical examinations at Murakami Memorial Hospital from 2004 to 2015. All participants completed a detailed questionnaire on their demographic characteristics and health behaviours. A trained staff member measured the demographic data, such as body weight and waist circumference (WC). Data on laboratory-related biochemical parameters were collected under standardised conditions and processed using a unified process. Since this was a retrospective cohort study, the risk of selection and observation biases was reduced.

The original research was approved by the ethics committee of Murakami Memorial Hospital, and informed consent was obtained from all participants. The authors of the original research handed over all copyrights of these data. Therefore, this study performed a secondary analysis based on their data without prejudice to the authors’ rights.

### Study sample

**Fig. 1:**
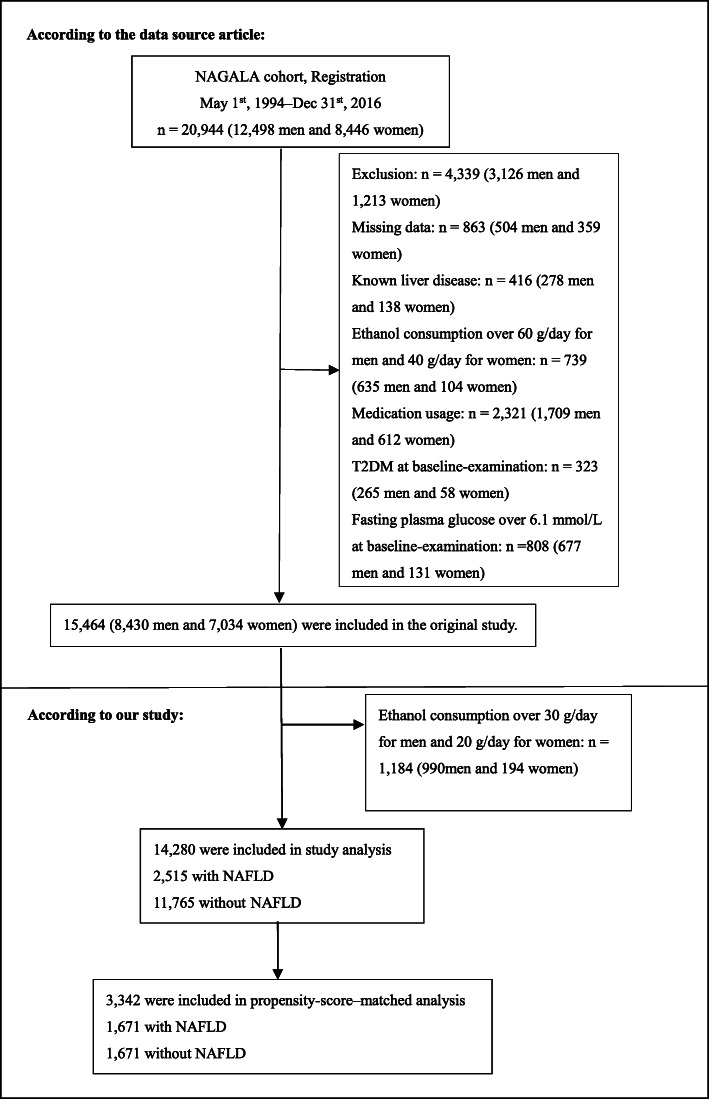
Study Population

In the original study, 5480 participants were excluded from 20,944 Japanese participants based on the following criteria: (1) viral hepatitis (defined by measurements of hepatitis B antigen and hepatitis C antibody at baseline), (2) alcoholic fatty liver disease, (3) DM at baseline, (4) fasting plasma glucose (FPG) level of ≥6.1 mmol/L, (5) use of any medication at baseline, and (6) missing covariate data. Therefore, 15,464 participants were included in the original study. This study further excluded 1184 participants with excessive alcohol consumption (alcohol consumption > 210 g/week in males and > 140 g/week in females [13]). Finally, this study included 14,280 eligible participants. Figure 1 detailed the selection process for all the participants.

### Independent variable and covariates

The independent variable was baseline NAFLD, which was diagnosed using abdominal ultrasonography performed by trained technicians [12]. The following covariates were extracted at baseline: age, gender, WC, body mass index (BMI), alcohol consumption, smoking status, regular exerciser, systolic blood pressure (SBP), diastolic blood pressure (DBP), aspartate aminotransferase (AST), alanine aminotransferase (ALT), total cholesterol (TC), gamma-glutamyl transferase (GGT), glycosylated haemoglobin A1c (HbA1c), FPG, high-density lipoprotein cholesterol (HDL-C), and triglycerides (TG). Alcohol consumption was classified into three categories: no or very little alcohol consumption (less than 40 g of alcohol per week), light alcohol consumption (40–140 g of alcohol per week), and moderate alcohol consumption (140–210 g of alcohol per week) [14]. Participants who regularly performed any type of exercise at least once a week were defined as regular exercisers [15]. Visceral fat obesity was defined as WC ≥ 90 cm in males or ≥ 80 cm in females [16].

### Outcome measure

The outcome was the incidence of DM. DM was defined as HbA1c ≥ 6.5%, FPG ≥ 7 mmol/L [17], or self-reported during follow-up.

### Statistical analyses

Continuous variables conforming to the normal distribution were presented as mean ± standard deviation (SD), while continuous variables conforming to the skewed distribution were expressed as median and quaternary ranges (25–75th percentile). Categorical variables were expressed as frequencies and percentages. The one-way ANOVA, the Kruskal-Wallis H test and the chi-square test were performed to detect differences between the groups. Missing values of HDL-C were handled by supplementing them with the mean.

PSM analysis was used to match the baseline characteristics between the NAFLD and non-NAFLD groups (Table 1), and to form a single group of participants with similar baseline characteristics. The non-parsimonious multivariable logistic regression model was performed to calculate the PS based on NAFLD as the independent variable and 17 baseline variables as covariates. This study used a 1:1 matching protocol without replacement (greedy matching algorithm), and the calliper width was equal to 0.01. The evaluation index of the balance between groups was the standardized differences [18, 19]. If the standardized differences were less than 10.0%, the covariates between the two groups were considered to be well balanced [18, 19]. Besides, the Kaplan-Meier method was used to assess the incidence of DM in each group, and the log-rank test was conducted to determine significance. *P* values were calculated for each pair of groups (total three comparisons: Low PS vs. Medium PS, Low PS vs. High PS, Medium PS vs. High PS), with Bonferroni correction [20]. The Cox proportional-hazards regression model was performed to explore the relationship between NAFLD and the incidence of DM by adjusting for covariates in the PSM cohort. The doubly robust estimation method, which combines PS models and the multivariate regression model, was applied to verify the association between NAFLD and the incidence of DM [21, 22]. Prespecified subgroup analyses were conducted based on gender, WC, BMI, AST, ALT, TC, GGT, HbA1c, FPG, HDL-C, TG, and PS. Specifically, continuous variables were converted to categorical variables based on the clinical cut-off point or median. Each stratification was adjusted for all factors, except for the stratification factor. In the subgroup analyses, only the corresponding matched pairs in the same subgroup were chosen to maintain the balance of baseline characteristics between the NAFLD and non-NAFLD groups. For example, in the subgroup of participants with BMI < 25 kg/m^2^, only when the matched pairs of the NAFLD and non-NAFLD groups both belonged to the BMI < 25 kg/m^2^ subgroup, these participants could be included in the subgroup analysis. Likelihood ratio tests were used to inspect the modifications and interactions of the subgroups.

**Table 1:** Baseline characteristics before and after propensity score matching

Before Matching				After Matching				
Characteristic	non-NAFLD	NAFLD	Standardized Difference (100%)	***P***	non-NAFLD	NAFLD	Standardized Difference (100%)	***P***
**Participants**	11,765	2515			1671	1671		
**Age (years)**	43.27 ± 8.99	44.78 ± 8.32	17.5	< 0.001	45.68 ± 9.14	45.47 ± 8.34	2.4	0.482
**Gender**			78.2	< 0.001			1.7	0.622
**Male**	5403 (45.92%)	2037 (80.99%)			1292 (77.32%)	1280 (76.60%)		
**Female**	6362 (54.08%)	478 (19.01%)			379 (22.68%)	391 (23.40%)		
**BMI (kg/m**^**2**^**)**	21.33 ± 2.61	25.50 ± 3.13	144.7	< 0.001	24.37 ± 2.61	24.40 ± 2.49	1.2	0.735
**WC (cm)**	74.10 ± 7.92	85.98 ± 7.78	151.3	< 0.001	83.17 ± 6.89	83.22 ± 6.41	0.8	0.810
**SBP (mmHg)**	111.93 ± 14.03	123.44 ± 14.83	79.7	< 0.001	120.45 ± 14.13	120.91 ± 14.28	3.2	0.349
**DBP (mmHg)**	69.71 ± 9.86	77.83 ± 10.19	81.0	< 0.001	75.77 ± 9.72	76.02 ± 9.65	2.6	0.454
**FPG (mg/dL)**	91.79 ± 7.24	97.19 ± 6.55	78.2	< 0.001	96.43 ± 6.62	96.34 ± 6.63	1.3	0.715
**HbA1c (%)**	5.15 ± 0.31	5.30 ± 0.33	46.4	< 0.001	5.26 ± 0.33	5.26 ± 0.33	1.1	0.746
**ALT(U/L)**	15 (12, 20)	27 (20, 39)	95.8	< 0.001	21 (16, 29)	24 (18, 31)	3.4	0.328
**AST(U/L)**	17 (14, 20)	20 (17, 26)	55.5	< 0.001	18 (15, 22)	19 (16, 23)	1.9	0.587
**GGT(U/L)**	14 (11, 18)	23 (16, 33)	61.5	< 0.001	19 (14, 28)	20 (15, 28)	1.0	0.781
**TC (mg/dL)**	195.50 ± 32.98	210.43 ± 33.55	44.9	< 0.001	207.09 ± 34.14	207.94 ± 33.42	2.5	0.464
**TG (mg/dL)**	58 (40, 84)	110 (77, 159)	95.8	< 0.001	92 (65, 132)	98 (70, 138)	3.7	0.284
**HDL-C (mg/dL)**	58.71 ± 15.33	45.87 ± 11.07	96.1	< 0.001	47.91 ± 12.48	47.98 ± 11.59	0.6	0.873
**Smoking status**			35.2	< 0.001			4.3	0.459
**Never smoker**	7565 (64.30%)	1186 (47.16%)			783 (46.86%)	811 (48.53%)		
**Ever smoker**	1930 (16.40%)	642 (25.53%)			417 (24.96%)	420 (25.13%)		
**Current smoker**	2270 (19.29%)	687 (27.32%)			471 (28.19%)	440 (26.33%)		
**Alcohol consumption**			3.2	0.332			8.7	0.042
**Non**	8887 (75.54%)	1888 (75.07%)			1185 (70.92%)	1215 (72.71%)		
**Light**	2302 (19.57%)	486 (19.32%)			388 (23.22%)	336 (20.11%)		
**Moderate**	576 (4.90%)	141 (5.61%)			98 (5.86%)	120 (7.18%)		
**Regular exerciser**			7.6	< 0.001			1.9	0.575
**NO**	9667 (82.17%)	2137 (84.97%)			1403 (83.96%)	1391 (83.24%)		
**YES**	2098 (17.83%)	378 (15.03%)			268 (16.04%)	280 (16.76%)		

Values were n (%) or mean ± SD or median (interquartile range: 25th to 75th percentiles)

*SD* standard deviation, *BMI* body mass index, *WC* waist circumference, *SBP* systolic blood pressure, *DBP* diastolic blood pressure, *FPG* fasting plasma glucose, *HbA1c* glycosylated haemoglobin, *ALT* alanine aminotransferase, *AST* aspartate aminotransferase, *GGT* gamma-glutamyl transferase, *TC* total cholesterol, *TG* triglyceride, *HDL-C* high-density lipoprotein cholesterol

For sensitivity analyses, the inverse probability of treatment weights (IPTW) was calculated using the estimated PS. For instance, the weight of NAFLD participants was 1/PS, and the weight of non-NAFLD participants was 1/(1 - PS). The IPTW model was conducted to create a weighted cohort [22]. In the sensitivity analysis, two association inference models were added to the original and weighted cohorts. A series of sensitivity analysis methods were used to test the robustness of the findings of the study and how conclusions could be affected by applying different association inference models. The effect sizes and *P-values* were calculated in all models. The results of this study were reported following the STROBE statement [23].

The current research analysis was performed using Empower-Stats (http://www.empowerstats.com, X&Y Solutions, Inc., Boston, MA) and the statistical software package R (http://www.R-project.org, The R Foundation). A two-sided *P* < 0.05 was considered significant.

## Results

### Study population

A total of 14,280 participants were eventually included in this study, including 52.10% men and 47.90% women (Fig. 1). Among them, 2515 (17.61%) participants suffered from NAFLD, and 11,765 (82.39%) did not suffer from NAFLD. The average age of the study population was 43.53 ± 8.89 years. During a mean follow-up of 2207.02 ± 1376.51 days, 324 participants developed DM. Some baseline characteristics showed statistically significant differences between the NAFLD and non-NAFLD groups before PSM. Higher levels of age, BMI, WC, SBP, DBP, FPG, HbA1c, AST, ALT, GGT, TC, and TG were observed in the NAFLD group. Participants with NAFLD showed a higher proportion of males, ever smoker, and current smoker. However, participants with non-NAFLD had higher HDL-C levels and higher rate of regular exerciser. In total, 1671 NAFLD patients were matched with 1671 non-NAFLD subjects by using one-to-one PSM. The standardized differences of all covariates were less than 10.0% after PSM, showing a good match. In other words, the differences in baseline characteristics between the two groups were minimal.

### The incidence of DM

**Fig. 2:**
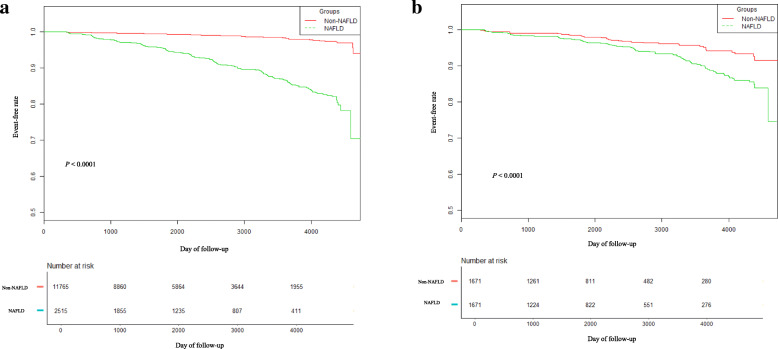
Kaplan–Meier event-free survival curve based on NAFLD and non-NAFLD **a** Kaplan–Meier analysis of incident diabetes based on NAFLD and non- NAFLD in the original cohort (log-rank, *P* < 0.0001). **b** Kaplan–Meier analysis of incident diabetes based on NAFLD and non-NAFLD in the propensity score matching cohort (log-rank, *P* < 0.0001)

**Fig. 3:**
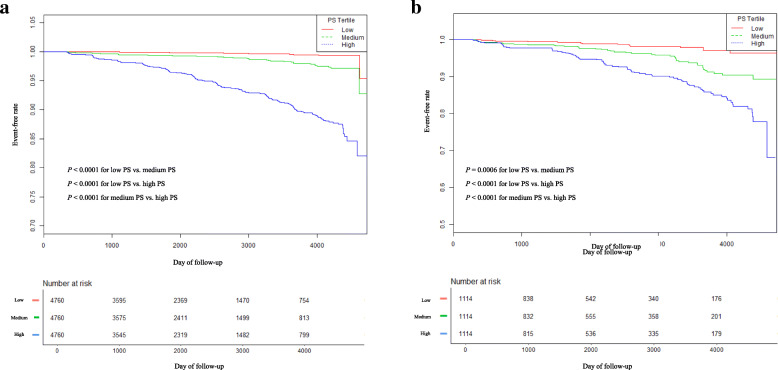
Kaplan–Meier event-free survival curve based on propensity score tertile. **a** Kaplan–Meier analysis of incident diabetes based on propensity score (PS) tertile in the original cohort (log-rank, *P* < 0.0001). *P* values were calculated for each pair of two groups (total three comparisons: Low PS vs. Medium PS, Low PS vs. High PS, Medium PS vs. High PS) with Bonferroni correction. **b** Kaplan–Meier analysis of incident diabetes based on propensity score (PS) tertile in the propensity score matching cohort (log-rank, *P* < 0.0001). *P* values were calculated for each pair of two groups (total three comparisons: Low PS vs. Medium PS, Low PS vs. High PS, Medium PS vs. High PS) with Bonferroni correction

The results of the Kaplan–Meier analysis revealed that the cumulative incidence of DM among the participants with NAFLD was significantly higher than that among participants without NAFLD before PSM (*P* < 0.0001; Fig. 2a). This difference still existed in the PSM cohort (*P* < 0.0001; Fig. 2b). Moreover, the cumulative incidence of DM was significantly higher in participants with higher PS after Bonferroni correction (Fig. 3).

### Association between NAFLD and the incidence of DM

The Cox proportional hazards regression model was applied to assess the association between NAFLD and DM risk in the PSM cohort. Table 2 showed the unadjusted, partially adjusted, fully adjusted, and propensity-score adjusted models. NAFLD was significantly associated with the incidence of DM in the unadjusted model. Participants with NAFLD were 98% more likely to develop DM (HR = 1.98, 95% CI: 1.41–2.80, *P* < 0.0001). The correlation still existed after adjusting for the partial confounding variables (age, gender, BMI, WC, smoking status, alcohol consumption, regular exerciser, SBP, DBP) (HR = 2.15, 95% CI: 1.52–3.04, *P* < 0.0001). In the fully adjusted model (adjusted for age, gender, BMI, WC, smoking status, alcohol consumption, regular exerciser, SBP, DBP, ALT, AST, GGT, HbA1c, FPG, TC, TG, and HDL-C), the association between NAFLD and the incidence of DM was still observed (HR = 2.33, 95% CI: 1.63–3.32, *P* < 0.0001). The risk of developing DM in the NAFLD participants was 2.33 times that of the non-NAFLD participants in the PSM cohort after adjusting for the demographic and laboratory biochemical variables. After adjusting for PS, the association was still observed, and participants with NAFLD had a 95% increased risk of DM (HR = 1.95, 95% CI: 1.39–2.75, *P* = 0.0001).

**Table 2:** Association between NAFLD and incident diabetes in different models

Variable	Non-adjusted (HR, 95% CI, *P*)	Model I (HR, 95% CI, *P*)	Model II (HR, 95% CI, *P*)	Model III (HR, 95% CI, *P*)
Non-NAFLD	Ref.	Ref.	Ref.	Ref.
NAFLD	1.98 (1.41, 2.80) < 0.0001	2.15 (1.52, 3.04) < 0.0001	2.33 (1.63, 3.32) < 0.0001	1.95 (1.39, 2.75) 0.0001

Crude model: we did not adjust for other covariates

Model I: we adjusted for age, gender, BMI, waist circumference, smoking status, alcohol consumption, regular exerciser, SBP, DBP

Model II: we adjusted for age, gender, BMI, waist circumference, smoking status, alcohol consumption, regular exerciser, SBP, DBP, ALT, AST, GGT, HbA1c, FPG, TC, TG, HDL-C

Model III: we adjusted for propensity score

*HR* Hazard ratios, *CI* Confidence interval, *Ref* Reference

### Subgroup analysis

Subgroup analysis was applied to discover potential confounding variables that might have affected the association between NAFLD and DM risk. Gender, BMI, WC, TC, TG, HDL-C, FPG, HbA1c, ALT, AST, GGT, and PS were chosen as stratification variables. Table 3 showed that none of the interactions were observed based on the prior specifications. The results revealed that the variables listed above did not affect the association between NAFLD and DM risk after PSM.

**Table 3:** Effect size of NAFLD on incident diabetes in prespecified and exploratory subgroups

Characteristic	No. of participants	HR (95% CI)	*P* value	*P* for interaction
Gender				0.2386
Male	2048	2.04 (1.32, 3.15)	0.0013	
Female	246	4.34 (0.29, 64.28)	0.2853	
BMI (kg/m^2^)				0.1157
< 25	1538	1.59 (0.87, 2.92)	0.1353	
≥ 25	662	3.40 (1.61, 7.17)	0.0013	
Visceral fat obesity				0.0688
NO	1996	1.67 (1.02, 2.73)	0.0410	
YES	272	4.75 (1.49, 15.11)	0.0083	
FPG (mg/dL)				0.2901
Low	758	5.31 (0.64, 44.16)	0.1221	
High	1056	1.88 (1.12, 3.15)	0.0172	
HbA1c (%)				0.2688
Low	494	11.99 (0.87, 164.39)	0.0629	
High	1258	1.95 (1.20, 3.20)	0.0076	
TC (mg/dL)				0.9720
Low	834	3.01 (1.33, 6.79)	0.0081	
High	852	2.82 (1.32, 6.04)	0.0076	
TG (mg/dL)				0.1982
Low	914	1.98 (0.76, 5.13)	0.1607	
High	938	4.10 (2.16, 7.77)	< 0.0001	
HDL-C (mg/dL)				0.8977
Low	892	1.96 (1.11, 3.46)	0.0197	
High	916	2.07 (0.86, 4.97)	0.1039	
ALT (U/L)				0.0611
Low	948	1.19 (0.55, 2.56)	0.6603	
High	968	2.82 (1.52, 5.25)	0.0011	
AST (U/L)				0.2397
Low	882	1.39 (0.63, 3.07)	0.4144	
High	902	2.65 (1.32, 5.32)	0.0060	
GGT (U/L)				0.2994
Low	880	1.97 (0.82, 4.73)	0.1294	
High	900	3.37 (1.80, 6.33)	0.0002	
Propensity score				0.3788
Low	1086	1.16 (0.36, 3.74)	0.8042	
Medium	1060	1.73 (0.88, 3.41)	0.1095	
High	1088	2.71 (1.68, 4.39)	< 0.0001	

Note 1: The above model has been adjusted for age, gender, BMI, waist circumference, smoking status, alcohol consumption, regular exerciser, SBP, DBP, ALT, AST, GGT, HbA1c, FPG, TC, TG, HDL-C

Note 2 In each case, the model was not adjusted for the stratification variable

### Sensitivity analysis

The estimated PS was used to generate a weighted cohort by establishing an IPTW model. This study evaluated the association between NAFLD and the incidence of DM in both the original and weighted cohorts. Moreover, the unadjusted, partially adjusted, and fully adjusted models were established in both cohorts (Table 4). The results demonstrated that NAFLD was significantly associated with the risk of DM in the original and weighted cohorts. In the fully adjusted models, the risk of developing DM in the NAFLD participants was 2.17 times and 2.27 times that of the non-NAFLD participants in the original cohort and weighted cohort (HR = 2.17, 95% CI: 1.63–2.88, *P* < 0.0001, HR = 2.27 95% CI: 1.91–2.69, *P* < 0.00001), respectively.

**Table 4:** Association between NAFLD and incident diabetes in different models of the original and the weighted cohort

Variable (A)	Non-adjusted	Model I (HR, 95% CI, *P*)	Model II (HR, 95% CI, *P*)
Non-NAFLD	Ref.	Ref.	Ref.
NAFLD	8.07 (6.44, 10.11) < 0.0001	3.79 (2.88, 4.98) < 0.0001	2.17 (1.63, 2.88) < 0.0001
Variable (B)	Non-adjusted	Model I (HR, 95% CI, *P*)	Model II (HR, 95% CI, *P*)
Non-NAFLD	Ref.	Ref.	Ref.
NAFLD	2.72 (2.31, 3.21) < 0.0001	2.61 (2.21, 3.08) < 0.0001	2.27 (1.91, 2.69) < 0.0001

A In the original cohort; B In the weighted cohort

Crude model: we did not adjust for other covariates

Model I: we adjusted for age, gender, BMI, waist circumference, smoking status, alcohol consumption, regular exerciser, SBP, DBP

Model II: we adjusted for age, gender, BMI, waist circumference, smoking status, alcohol consumption, regular exerciser, SBP, DBP, ALT, AST, GGT, HbA1c, FPG, TC, TG, HDL-C

*HR* Hazard ratios, *CI* Confidence interval, *Ref* Reference

## Discussion

The PSM cohort study showed that NAFLD was an independent risk factor for the development of DM. The risk of developing DM in the NAFLD participants was 2.33 times that of the non-NAFLD participants in the PSM cohort after adjusting for the demographic and laboratory biochemical variables. This figure decreased to 95% after adjusting for the PS. In the subgroup analysis, no interaction was observed, indicating that the relationship between NAFLD and DM was robust. The correlation also existed in both the original and weighted cohorts.

NAFLD can develop into liver fibrosis, cirrhosis, and liver cancer and increase the risk of developing diabetes and cardiovascular diseases [24]. Patients with NAFLD have been reported to have a higher prevalence of prediabetes/DM and increased IR [9, 25]. The incidence of DM in NAFLD patients was also higher in NAFLD participants than in non-NAFLD patients, even if their plasma glucose levels were within normal ranges [26]. The improvement of NAFLD was related to a decrease in the incidence of DM [27]. It has been reported that NAFLD and DM have some same risk factors and often occur simultaneously in one person [6, 10]. Meanwhile, several prospective studies have found that NAFLD strongly increases the incidence of DM [28, 29]. In addition, a study explored the relationship between NAFLD and DM using PSM methods [30]. Their findings suggested that NAFLD was a risk factor for DM, which was consistent with the conclusion of this study. However, that study excluded participants with other metabolic diseases (hypertension, dyslipidaemia), and NAFLD was mainly diagnosed by non-invasive scores [30]. Based on these findings, the prevalence of NAFLD might be underestimated. Therefore, the results of the study mentioned above could not be applied to the general population. In this study, NAFLD was diagnosed by abdominal ultrasonography, and the study population was more extensive. These results could better reflect the actual relationship between NAFLD and DM. In contrast, some studies showed different findings. They showed that the association between NAFLD and the risk of DM was not significant after adjusting for confounding factors [31, 32]. The possible reasons for these inconsistent findings were as follows: (1) The study population was diverse, including different races, genders, ethnicities, ages, and so on. (2) The sample size varied greatly between the different studies. (3) These studies adjusted for different confounding variables, which affected the relationship between NAFLD and DM. (4) Due to the difference in the follow-up time, the incidence of DM varied widely. This research strongly supports the results of existing studies that NAFLD increases the risk of developing DM.

In this large-scale cohort study, the risk of developing DM in the NAFLD participants was 2.33 times that of the non-NAFLD participants after PSM. There was a difference between this study and previous studies in terms of the risk of DM, which might be related to the fact that this study conducted a PSM analysis and effectively controlled for more confounding variables which were well known to be related to NAFLD and DM, including age, gender, BMI, WC, smoking status, alcohol consumption, regular exerciser, SBP, DBP, ALT, AST, GGT, HbA1c, FPG, TC, TG, and HDL-C [8, 33]. Additionally, this study was based on large cohort data (14,280 participants), which further strengthened the statistical power of the results. Exploring the association between NAFLD and DM can help us better guide patients in clinical practice and develop management strategies to reduce DM risk [34, 35]..

The mechanism by which NAFLD leads to DM remains unclear. A study demonstrated that NAFLD could cause IR, which could further mediate the development of DM [36]. The mechanisms by which NAFLD contributes to IR are as follows: (1) Adipose tissue dysfunction and inflammation promote the secretion of adipokines, increase the secretion of pro-inflammatory factors (such as tumor necrosis factor-α), and increase the release of free fatty acids, resulting in decreased insulin sensitivity. Adipose tissue dysfunction and inflammation interfere with the activation of the pro-inflammatory pathway of insulin signal transmission, leading to decreased insulin sensitivity [37]. (2) Certain incretin related to NAFLD can directly inhibit the production of endogenous glucose through an insulin-dependent mechanism [38]. The reduction of these incretin effects also leads to IR [38]. (3) Increased expression of dipeptidyl peptidase-4 impairs insulin sensitivity by reducing incretin levels and promoting liver disease progression through independent mechanisms [39, 40].

### Study strengths and limitations

This study has the following strengths. The most innovative part of this study is that PSM was used to explore the relationship between NAFLD and the risk of developing DM. In recent years, the PSM method has been widely used in observational research. The acknowledged advantages of the PSM method include a wide range of data requirements, including a reduction of inter-group differences, balancing inter-group confounders, and achieving the effect of “similar randomization”. Subgroup analyses were performed to explore other potential risk factors that could affect the association between NAFLD and DM. A series of sensitivity analyses were conducted to ensure the robustness of the results. This study mainly used IPTW to establish a weighted cohort and further explore the association between NAFLD and the incidence of DM in the weighted cohort. More importantly, the sample size of the participants in this study was more extensive than that in most previous retrospective cohort studies.

However, the current study has several limitations. First, the population included in this study was Japanese, and therefore, the generalizability of these results to other races requires further validation. Second, the lack of a 2-h oral glucose tolerance test in the original study might have underestimated the incidence of DM. However, it is not feasible to conduct a 2-h oral glucose tolerance test in such a large cohort. Third, the PSM could balance known confounding variables as much as possible, but it could not ensure that all measured baseline characteristics were matched and consider the influence of unknown variables. To reduce the interference of variables on the measurement results, the calliper width was set at 0.01. Fourth, ultrasonography may have some limitations in diagnosing NAFLD. However, some non-invasive scores, such as the FIB4 score, have some advantages. Considering that the original data lack relevant data, such as platelets, FIB4 scores could not be used to diagnose NAFLD. In the future, it would be worthwhile to design studies or collaborate with other researchers to collect as many variables as possible to analyse the actual relationship between the non-invasive score of NAFLD and DM. Fifth, the differences between type 1 and type 2 DM were not considered in the present study. However, type 2 DM is most common, accounting for over 90% of the cases of DM [41]. Therefore, this study aimed to explore the relationship between NAFLD and type 2 DM.

## Conclusions

NAFLD was an independent risk factor for the development of DM. After adjusting for the demographic and laboratory biochemical variables, the risk of developing DM in the NAFLD participants was 2.33 times that of the non-NAFLD participants in the PSM cohort. The participants with NAFLD had a 95% increased risk of DM after adjusting for PS.

## Data Availability

The data are available from the ‘DataDryad’ database (www.datadryad.org).

## Abbreviations

BMI: Body mass index
WC: Waist circumference
SBP: Systolic blood pressure
DBP: Diastolic blood pressure
FPG: Fasting plasma glucose
HbA1c: Glycosylated haemoglobin A1c
ALT: Alanine aminotransferase
AST: Aspartate aminotransferase
GGT: Gamma-glutamyl transferase
TC: Total cholesterol
TG: Triglyceride
HDL-C: High-density lipoprotein cholesterol
T2DM: Type 2 diabetes mellitus
DM: Diabetes mellitus
SD: Standard deviation
HR: Hazard ratios
CI: Confidence intervals
Ref: Reference
PS: Propensity score
IPTW: Inverse probability of treatment weights
NAFLD: Nonalcoholic fatty liver disease
IR: Insulin resistance
